# A Reply to the Criticism of Dr. Holmes

**Published:** 1889-02

**Authors:** H. P. Holmes


### A REPLY TO THE CRITICISM OF DR. HOLMES.

Editors Homœopathic Physician:—In the December number of your excellent journal I find a criticism of myself, and have been asked by postal card and letter from the author to reply to it. This is the first time I have ever been called upon to enter into a controversy through the columns of a journal. I shrink from imposing such an uncalled-for infliction upon the readers of a medical publication, and would rather let the criticism go unanswered, if I were certain this erudite gentleman would not do the same thing over again the first opportunity that offers.

That I may be plainly understood in this matter, and my position exactly shown to all, I will go back to the original statement which has caused all this discussion, viz.: "*I think a great error in our method of prescribing is to memorize the materia medica. I have made it a point never to memorize any remedy, and I do not believe I could give you the characteristics of a dozen remedies.*" The advice given as to the better way was to be so armed with books as to be able to study the remedy up at once, and so prescribe accurately. To be certain that I had not misunderstood the statement, I asked a question, and the answer and the following discussions clenched the method as I had understood it.

This method struck a killing blow at one of my long-cherished ambitions, and one that I have struggled harder for than any other thing in my medical practice—that of being a brilliant prescriber. Not that I may ever attain that reputation, but I should like to. In the discussion which followed I am willing to admit that many of us said things we did not mean literally, and which had little bearing on the question. But as for myself, I was sincere in my objection to the above statement in its *literal* meaning as given and emphasized.

For the stand I took I have been pretty roughly handled; have been called, inferentially and directly, a *mongrel, conceited ass*, and *fool;* have been told by my able criticiser that my language was *offensive*, that I was *negligent* and *lazy*, and that a certain inferred confession was *criminal.* Now, I will ask any fair-minded person if it is not utterly hopeless to attempt to sincerely discuss a question with such a peculiarly-minded person as is plainly manifested by the above epithets? If, in order to be considered a strict Hahnemannian, it is necessary to resort to blackguarding my opponents by way of argumentative intelligence, then I must say I wish to withdraw from the controversy. If he wishes to discuss this question in a serious, candid manner, with the good of our grand school in view, and the wish to help one who is honestly trying to better himself in his professional standing, then I am with him.

My article in your November issue was written in a spirit of fun to show my position by stating a perfectly simple case where the remedy was plainly indicated, and where one could easily prescribe the correct remedy without resorting to repertories, provided he had anything like a smattering of the characteristics of some of the leading remedies. It was only in these simple cases that I asked the right to prescribe off-hand. There was no idea of insulting any one, and no one, unless he has a disordered liver, will think of taking such a malicious view of it as has my would-be director.

Well, my criticiser fell into that little thing all over. As Uncle Remus said of Brer Rabbit when the pail of honey fell on him, "he wa'n't dess only bedobble wid it, he was dess kiver'd." He has drawn all sorts of false inferences from my report, and then with remarkable boldness has gone to work and thoroughly demolished the vagaries of his own disordered fancy. It is impossible to find out just what he is driving at. He admits that the prescription was a good one, and then says, "we have no evidence that the prescription cured." The gentleman should have inclosed this remark with quotation marks, as the old school have been using it against all homœopaths for nearly a century, and the so-called "mongrels" against Hahnemannians for nearly the same length of time. It is hard for some men to forget the influence of their early teaching. With the above illiberal spirit what *can* we hope from such a man? The peculiarity of his position will be more apparent when I say that I wrote to the honorable gentleman begging him not to make this a personal controversy, and that he perfectly understands my position. He writes me that he does not use a book more than three or four times in making forty prescriptions. Why did he not say so last summer, and so put himself in a true light? His inferences regarding my non-use of books is also overdrawn and wilfully untrue, for I had written him just how and when I used them, and he virtually admitted that our methods were the same. When a man in his position thinks there is any physician in active practice who does not study his difficult cases until heart and brain grow sick and weary with the responsibility and labor, it shows him sadly wanting in that broad charity so necessary to be shown toward his brother physicians.

The most glaring fault in his paper, from a literary standpoint, and the first one to catch the reader's eye, is the continual repetition of my name. Ten times he enters upon the unnecessary task of spelling it out in full, and for a wonder gets it right every time. If a would-be instructor makes such an egregious blunder in so short a controversial article, what must we think of his other abilities? It would seem as if his ignorance of the most common rule of discussion must be taken as an evidence of the depth of his abilities in his professional teachings.

He questions my right to leave six powders to be given every half hour, if necessary, instead of relying upon one. If the gentleman will turn to the *Organon*, paragraph 247 (and by the way, he is lecturing on the *Organon*), he will find my authority for the frequent repetition of the dose, viz.: "In the most acute diseases, at intervals varying from an hour to five minutes." It must be exceedingly irritating to the shade of Hahnemann to know that our Philadelphia friend does not agree with him in this paragraph, and even criticises an earnest disciple of Homœopathy who is guided in his practice by the above plain instruction. Well, it is to me too!

If this gentleman would have a broader, more liberal view of his profession, more charity for his fellow-workers, and more respect for their methods, their beliefs, and their abilities, it would be better for him and certainly better for the great school he represents. It would place him less in the light of a homœopathic anarchist.*

H. P. Holmes.

* We cannot see any advantage to be derived from such personal discussions, therefore we hope this one will end with Dr. Holmes's answer.—Editors Homœopathic Physician.

